# Five Things to Know About Incremental Peritoneal Dialysis

**DOI:** 10.1177/20543581231192748

**Published:** 2023-08-11

**Authors:** Mohammed Azfar Qureshi, Shabnam Hamidi, Bourne L. Auguste

**Affiliations:** 1 Department of Medicine, University of Toronto, ON, Canada; 2 Division of Nephrology, Sunnybrook Health Sciences Centre, Toronto, ON, Canada; 3 Centre for Quality Improvement and Patient Safety, University of Toronto, ON, Canada

**Keywords:** incremental peritoneal dialysis, solute clearance, patient-centered care, shared decision-making

## Abstract

Incremental peritoneal dialysis (PD) offers patients newly starting dialysis less than the standard “full dose” of PD, reducing treatment burden and intrusiveness while minimizing symptoms of renal failure. Incremental PD is a cost-effective approach that has been associated with slower rates of decline in residual kidney function. This approach also produces less waste and in turn reduces environmental footprint compared to standard PD prescriptions. It also aligns with the International Society of Peritoneal Dialysis (ISPD) Practice Recommendations for high-quality, goal-oriented therapy. Awareness of incremental PD along with its advantages and limitations provides practitioners with the tools to provide more patient-centered dialysis prescriptions in appropriate populations.

## Abrege

La dialyse péritonéale (DP) incrémentale propose un traitement à une dose moindre que la « dose complète » habituelle aux patients qui amorcent la dialyse; ce qui contribue à réduire le fardeau du traitement et à en limiter le caractère intrusif, tout en minimisant les symptômes de l’insuffisance rénale. La DP incrémentale est une approche qui présente un bon rapport coût/efficacité, en plus d’avoir été associée à un ralentissement du déclin de la fonction rénale résiduelle. Elle produit également moins de déchets que la DP standard, ce qui, par conséquent, réduit l’empreinte environnementale du traitement. Enfin, la DP incrémentale est conforme aux recommandations de pratique de l’International Society of Peritoneal Dialysis (ISPD) pour une thérapie de haute qualité axée sur les objectifs. La sensibilisation à la DP incrémentale, ainsi qu’à ses avantages et à ses limites, fournit aux praticiens les outils nécessaires pour prescrire une modalité de dialyse davantage centrée sur le patient dans les populations appropriées.

## Incremental Peritoneal Dialysis is an Effective Treatment Strategy That Focuses on Goal-Directed Therapy for Incident Patients on Dialysis

**Figure 1.**
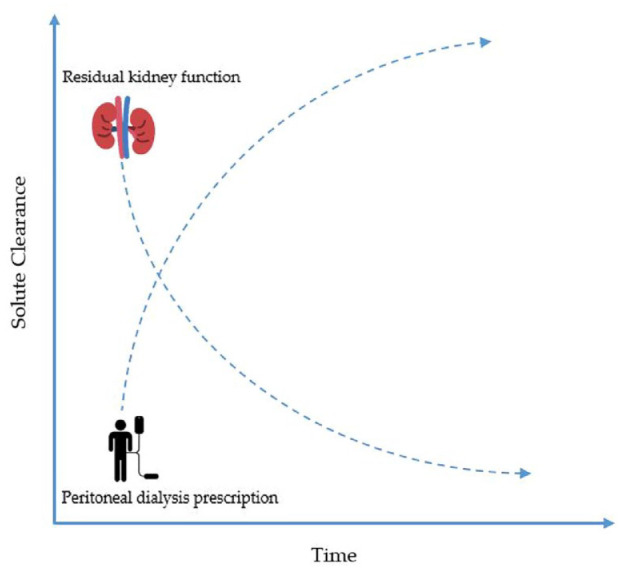
As residual kidney function declines with time, the peritoneal dialysis prescription is increased incrementally to improve solute clearance.

The initiation of dialysis treatments can be a significant burden for patients, family, and caregivers. Incremental peritoneal dialysis (PD) is a treatment strategy that offers less than the standard “full dose” of PD such that peritoneal clearance in combination with residual kidney function (RKF) leads to sufficient small solute clearance that reduces the burden of uremic symptoms.^1-3^ The PD prescription is ultimately increased as the RKF declines with time (Figure 1). This offers patients a gentle prescription when starting dialysis and can allow for smoother transition by offering individualized treatment with more flexibility in daily routines. This lowers the burden of intrusiveness that incident patients may experience when starting dialysis with improved quality of life (Table 1). Incremental PD also aligns with the 2020 International Society of Peritoneal Dialysis (ISPD) Practice Recommendations of prescribing high-quality goal-oriented dialysis treatments by offering PD that reduces treatment burden while minimizing symptoms through the provision of high-quality care.^4^ Shared decision-making and collaboration between the health care team and the patient are the key to success when initiating this strategy. The goal is to provide patient-centered care that optimizes outcomes and enhances quality of life for individuals starting dialysis. Therefore, incremental PD can be utilized to achieve realistic care goals for patients who are new to dialysis treatment.

**Table 1.** The Advantages and Disadvantages Associated with Incremental Peritoneal Dialysis Prescriptive Strategies.

Advantages	Drawbacks
Gradual transition to full dose PD	Not ideal for patients with significant volume overload needing large ultrafiltration volumes
Better preservation of RKF	Increased risk of under-dialysis if RKF declines and no changes are made with prescription
Reduced risk of mechanical complications	Need for frequent evaluation of prescription
Improved patient adherence and quality of life	Risk of therapeutic inertia on the part of patients
Reduced health care cost and environmental burden	Need for closer clinical monitoring and more regular follow-up
Reduced peritoneal membrane and systemic glucose exposure	Not ideal for acute start peritoneal dialysis

Source: Adapted with modification from Cheetham et al.^5^

PD = peritoneal dialysis; RKF = residual kidney function.

## Patients Managed with Incremental Peritoneal Dialysis Have Demonstrated Slower Rates of Decline in Residual Kidney Function Compared to Full-Dose Peritoneal Dialysis

The preservation of RKF is associated with better patient survival and improved quality of life in PD.^6^ Observational data have shown better preservation of RKF with incremental PD compared to full-dose PD.^7^ It has been postulated that the preservation in RKF seen with incremental PD is due to lower risk of hypotension by nature of this gentler dialysis prescription. In addition, the intact nephron hypothesis has also been highlighted as a possible mechanism of how incremental PD might preserve RKF by reducing hyperfiltration in remaining nephrons.^8,9^ Given the strong association between preserving RKF and patient survival, practitioners should consider adopting incremental PD practices where feasible.

## Adoption of Incremental Peritoneal Dialysis may be Associated with Lower Cost to the Health Care System with a Reduced Environmental Footprint

Observational data from Italy have revealed that incremental PD is associated with lower hospitalization rates and lower costs.^10^ Although formal studies examining the cost-effectiveness of incremental PD are lacking, experiences in a single Canadian center demonstrated that prescribing dialysis to achieve Kt/V adequacy targets resulted in a 16% annual increase in cost per patient.^11^ In adopting an incremental approach to PD prescription, the costs incurred to the health care system would be reduced because less dialysate solutions are being utilized. This leads to reduced waste-product disposal which also aligns with green kidney care and sustainable nephrology action planning initiatives.^12,13^ Cheetham and colleagues have also highlighted that reduced frequency in delivery of supplies with an incremental approach may lower carbon emissions and plastic waste from supplies.^5^ As a result, practitioners should consider the adoption of incremental PD in practice given that it can be more cost-effective with a reduced environmental footprint compared to standard PD.

## Patients Receiving Incremental Prescriptions Have Reduced Peritoneal Glucose Exposure and are at Lower Risk of Mechanical Complications on Peritoneal Dialysis

In incremental PD, patients are initially treated with lower volumes of dextrose-based dialysate solutions which in turn reduces peritoneal glucose exposure. This has been supported by a small randomized controlled trial in China that revealed a statistically significant difference in glucose exposure between standard and incremental PD prescriptions.^14^ In addition, high glucose exposure may lead to systemic complications such as hyperglycemia and weight gain from high caloric loads along with the development of glucose-degradation products which can be harmful to the peritoneal membrane.^15,16^ As it relates to mechanical complications, an incremental strategy that utilizes lower dwell volumes will result in lower intraperitoneal abdominal pressure and lead to less mechanical complications and discomfort for the patients.^1^ In utilizing a lower fill volume, practitioners can allow patients time to adapt to the fill volumes and gradually increase over time, allowing patients to become more comfortable with the treatment.

## Although the Prescription for Incremental Peritoneal Dialysis can be Provided in Different Ways, not all Patients are Suitable for This Treatment Strategy

Practitioners should be aware that there is no one specific way to provide patients with an incremental PD prescription. An incremental PD prescription can be done with continuous ambulatory PD or automated PD (Table 2). Patients can be started on 2 to 3 exchanges with either type of PD with fill volumes of 1.2 to 1.5 L per exchange.^1,2,17^ Patients can also have 1 to 2 days off as an alternative incremental approach.^1,17^ Similarly, patients with cardiorenal syndrome who start dialysis mainly for ultrafiltration purposes can also have an incremental approach. The incremental PD prescription chosen should ultimately align with patient priorities that ultimately fosters shared decision-making. However, in cases where patients have minimal or no RKF at the beginning or those on hemodialysis (HD) without RKF being transitioned to PD, they may not be well-suited for incremental PD. There are limited data to indicate the ideal estimated glomerular filtration rate (GFR) when incremental dialysis should be considered. The initiation of dialysis should be guided by a thorough clinical assessment of patients and evaluation of symptoms that would benefit from dialysis treatment and not arbitrary GFR cut-off values. It is important for practitioners to recognize that providing less dialysis compared to standard PD prescription may lead to suboptimal small solute clearance.^1,3,5^ This could result in under-dialysis in patients who are not ideal candidates for this approach. Observational data have demonstrated that a significant proportion of patients on incremental PD experienced episodes of under-dialysis, particularly those with lower baseline RKF.^18^ Given lack of robust data, if incremental PD is being utilized, patients must be followed closely for any significant change in RKF or change in clinical status. Practitioners must also inform patients that changes in PD prescription will be required with a decline in RKF. This pre-emptive counseling is essential in reducing the likelihood of “therapeutic inertia” on the part of patients when a change in prescription is needed.

**Table 2.** Examples of Incremental vs Standard PD Prescription.

Incremental PD prescription	Standard PD prescription
CAPD	
Two to three exchanges/day with 1 to 2 days off treatment	Four exchanges/day 7 days a week
One short exchange with 1 long dwell of icodextrin	Three short exchanges with 1 long dwell of icodextrin
APD	
Six hours 2 exchanges and day dry with 1 to 2 days off treatment	Eight to 9 hours 4 exchanges and day dry 7 days a week
Six hours 2 exchanges 1.5 L fill volume 7 days a week	Eight hours 4 exchanges 2 L fill volume with last fill using icodextrin 1.5 L fill volume

APD = automated peritoneal dialysis; CAPD = continuous ambulatory peritoneal dialysis; PD = peritoneal dialysis.
